# Ritz Solution of Vibration Analysis of Functionally Graded Porous Elliptic Shells and Panels Under Various Arbitrary Boundary Types

**DOI:** 10.3390/ma18051101

**Published:** 2025-02-28

**Authors:** Qingtao Gong, Tao Liu, Yao Teng, Binjie Ma, Xin Li

**Affiliations:** 1 Ulsan Ship and Ocean College, Ludong University, Yantai 264025, Chinatengyao@ldu.edu.cn (Y.T.); mabinjie@ldu.edu.cn (B.M.); lixin@ldu.edu.cn (X.L.); 2 Shandong Marine Aerospace Technology Innovation Center, Yantai 264025, China; 3 School of Civil Engineering, Central South University, Changsha 410075, China

**Keywords:** Ritz variational, functionally graded porous elliptic shells and panels, free vibration, Jacobi orthogonal polynomials

## Abstract

This paper seeks to establish a generalized numerical model to examine the free vibration behavior of functionally graded porous (FGP) elliptical shells and panels with various boundary types. The model is built on first-order shear deformation theory (FSDT) to express structural displacements. A segmentation technique is used to maintain continuity between shell elements, and virtual spring boundary techniques are employed to simulate arbitrary boundaries. Variable-coefficient Jacobi polynomials are introduced as admissible functions for displacement. Finally, the Ritz variational method, combined with the least-squares weighted residual method (LSWRM), is used for constructing the energy functional and solving the energy equations. Validation of the numerical model against finite element and literature results confirms its reliability and convergence properties. This study also explores the effects of geometric parameters and boundary conditions on FG elliptical shells and panels, providing a theoretical basis for future research.

## 1. Introduction

Elliptical shell structures are extensively applied in aerospace, marine engineering, and other fields [1,2,3]. FGP structures are a major focus of research for structural and materials engineers today [4,5,6]. Due to their unique distribution of material properties, these structures offer high specific strength and lightweight characteristics, making them especially suitable for large-scale engineering applications such as aerospace, marine vessels, and nuclear reactors. Current research shows that dynamic studies on new lightweight thin-walled structures combining elliptical shells with FGP materials are relatively scarce. Therefore, developing a unified dynamic model is essential for performing a comprehensive analysis of this structure.

Chen et al. [4,7,8] conducted extensive research on FGP beams, starting from Timoshenko beam theory and using the Ritz method to solve the energy equations, thereby comprehensively analyzing the bending and localized characteristics of the structure. Similarly, Kitipornchai et al. [6] used this approach to perform an analysis of the dynamic behavior of porous nanocomposite beams. For large-amplitude nonlinear vibrations, Ebrahimi and Zia [9] employed a multi-scale approach (MSA) combined with Galerkin’s method. Mojahedin et al. [10] studied the buckling characteristics of FGP circular plates using a higher-order shear deformation theory (HSDT), although the boundary conditions were not standard, and the computation speed was relatively slow. Rezaei et al. [11,12] used a simplified four-variable plate theory to derive analytical solutions for the frequencies of FGP rectangular plates. Additionally, his team [13,14,15] utilized Mindlin plate theory to investigate the vibration characteristics of moderately thick FG sector and annular plates, emphasizing porosity control techniques and examining how porosity impacts structural vibration behavior. Zenkour [16] used an advanced HSDT, known as the quasi-3D shear deformation theory, to study the bending vibrations of rectangular plates. This theory, now widely used for analyzing thick structures, offers higher computational efficiency compared to traditional HSDT. Wang and colleagues [17], skilled in using the Ritz method for vibration analysis, conducted in-depth research on the free vibration of general rotating FGP structures with various elastic boundaries. Belica [18], using the Bubnov–Galerkin method, investigated the vibration characteristics of porous honeycomb cylindrical shells under several simple support conditions. Gao [19] used the MSA to obtain an analytical solution for the nonlinear vibrations of FGP cylindrical shells, employing Donnell shell theory (DST) to construct the structural displacement expressions. He further examined the effects of a uniformly applied harmonic load, accounting for damping influences, and discovered that the type of FGP distribution plays a critical role in shaping the primary resonance response. Wang [20], using the Ritz method, analyzed the natural frequency of FGP cylindrical shells under clamped, simply supported, and free boundary conditions. Similarly, Zhao et al. [21,22,23,24] applied this approach to study the vibration characteristics of FGP beams and rectangular plates.

The preceding review highlights that most existing research on FGP shell structures primarily focuses on cylindrical shells, while studies on elliptical structures remain limited. To address this gap, this paper aims to develop a unified dynamic model for analyzing the free vibration behavior of FGP elliptical shells and panels under arbitrary boundary conditions. The motion equations at various points in the structure are derived based on the FSDT. The boundaries are modeled as a spring system with variable stiffness, which is combined with the structural stiffness matrix to achieve arbitrary boundary conditions. By means of segmentation technology, *N_L_* and *N_θ_* segments are separated along the axis and circumference of the FG elliptic shells. the total number of segments being *N_L_* × *N_θ_*. Building on this foundation, the modified variational principle (MVP) and LSWRM are applied to develop the energy functional for each segment, ensuring natural continuity across the segmented elements. Using this approach, the complexity of formulating the displacement functions of each segment is simplified, as the boundary conditions for individual segments can be treated as fully free. The displacement functions are constructed with Jacobi orthogonal polynomials to meet admissibility requirements. By applying variational principles, a global equation describing the free vibration behavior of FGP structures is formulated. Finally, the effectiveness of the method is confirmed through numerical analysis, providing a set of benchmark results that may support future research in this area.

## 2. Theoretical Equations

### 2.1. Overview of the FGP Model

**Figure 1:**
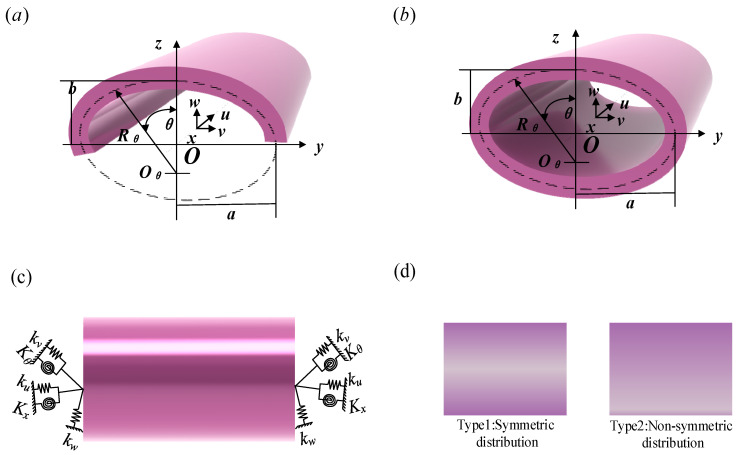
Structural schematic diagram and material cross-section diagram of FGP elliptical shells and panels.

A three-dimensional description of the FGP elliptic panels is shown in Figure 1a. The orthogonal coordinate system (*x*, *θ*, *z*) is anchored at the panel’s mid-surface, with *u*, *v*, and *w* representing the respective displacement directions. The geometric parameter symbols of the elliptic panels are defined as follows: thickness: *h*, length: *L*, circumferential angle: *θ*_0_, length of the major axis: *a*, length of the minor axis: *b*, radius of the elliptical shell’s midsection: *R_x_*(*θ*) and *R_θ_*(*θ*). It should be pointed out here that when the circumferential angle of the elliptic panels is 360 degrees, the elliptic panels will degenerate into a elliptic shells, as shown in Figure 1b. The boundary types are modeled using five sets of virtual springs, depicted in Figure 1c. Additionally, two porosity distribution types—symmetric and asymmetric—are considered along the thickness direction, as shown in Figure 1d. According to the typical mechanical properties of open-cell metal foam, the specific expressions for Young’s modulus *E*, shear modulus *G*, and mass density *ρ* are as follows [25]:

(1) $$\mathrm{Type}1\left\{\begin{array}{c} E\left(z\right)=e_{1}E_{1}\\ G\left(z\right)=e_{1}G_{1}\\ \rho \left(z\right)=e_{1}\rho_{1} \end{array}\right.,\begin{array}{ccc} & & e_{1}=1-e_{0}\cos\left(\frac{\pi z}{h}\right) \end{array}$$

(2) $$\mathrm{Type}2\left\{\begin{array}{c} E\left(z\right)=e_{2}E_{2}\\ G\left(z\right)=e_{2}G_{2}\\ \rho \left(z\right)=e_{2}\rho_{2} \end{array}\right.,\begin{array}{ccc} & & e_{2}=1-e_{0}\cos\left(\frac{\pi z}{2h}+\frac{\pi }{4}\right) \end{array}$$

where *e* is the porosity coefficient *e*_0_:

(3) $$e_{0}=1-E_{2}/E_{1}=1-G_{2}/G_{1},0\leq e_{0}\leq 1$$

### 2.2. Modified Variational Formulation Model

The FGP elliptic shells and panels are separated into *N_L_ × N_θ_* segments by means of segmentation technology. For each segment, all its boundary conditions are free boundaries. Within the framework of FSDT, the energy functional of each segment is readily achieved. The next challenge is to enforce continuity conditions at the internal interfaces between the segments. In this paper, the continuity constraints on the internal interfaces are achieved by using the Lagrange multipliers method. Based on MVP, the modified variational functional Π of the functionally graded (FG) porous elliptic panels can be expressed as follows [26]:

(4) $$\prod =\int_{t_{0}}^{t_{1}}\sum_{i=1}^{N_{L}}\sum_{j=1}^{N_{\theta }}\left(W_{i,j}+T_{i,j}-U_{i,j}\right)dt+\int_{t_{0}}^{t_{1}}\left(\sum_{i,i+1}{\tilde{\prod }}_{\lambda }+\sum_{j,j+1}{\tilde{\prod }}_{\gamma }\right)dt+\int_{t_{0}}^{t_{1}}\prod_{BC}dt$$

where indices (*i*, *j*) represent the segment numbers. *T*, *U*, and *W* denote the kinetic energy, potential energy, and work performed by external forces, respectively. It is worth mentioning that in the above equation, ${\tilde{\prod }}_{\lambda x}$ and ${\tilde{\prod }}_{\lambda \theta }$ are the variational function terms. Their role is to ensure the continuity in the two integration directions. The last term represents the energy integral of the boundary spring stiffness, which is the summation of the integrals of the five springs along their respective displacement directions. Their specific expressions are as follows:

(5) $$\prod =\int_{t_{0}}^{t_{1}}\sum_{i=1}^{N_{L}}\sum_{j=1}^{N_{\theta }}\left(W_{i,j}+T_{i,j}-U_{i,j}\right)dt+\int_{t_{0}}^{t_{1}}\left(\sum_{i,i+1}{\tilde{\prod }}_{\lambda }+\sum_{j,j+1}{\tilde{\prod }}_{\gamma }\right)dt+\int_{t_{0}}^{t_{1}}\prod_{BC}dt$$

(6) $${\tilde{\prod }}_{\lambda x}=\int_{l}\left(N_{x}\gamma_{u}+N_{x\theta }\gamma_{v}+Q_{x}\gamma_{w}+M_{x}\gamma_{x}+M_{x\theta }\gamma_{\theta }\right)dl$$

(7) $$\begin{array}{l} \gamma_{u}=u_{0}^{i,j}-u_{0}^{i+1,j},\gamma_{v}=v_{0}^{i,j}-v_{0}^{i+1,j},\gamma_{w}=w_{0}^{i,j}-w_{0}^{i+1,j},\gamma_{x}=\psi_{x}^{i,j}-\psi_{x}^{i+1,j},\gamma_{\theta }=\psi_{\theta }^{i,j}-\psi_{\theta }^{i+1,j}\\ \zeta_{u}=u_{0}^{i,j}-u_{0}^{i,j+1},\zeta_{v}=v_{0}^{i,j}-v_{0}^{i,j+1},\zeta_{w}=w_{0}^{i,j}-w_{0}^{i,j+1},\zeta_{x}=\psi_{x}^{i,j}-\psi_{x}^{i,j+1},\zeta_{\theta }=\psi_{\theta }^{i,j}-\psi_{\theta }^{i,j+1} \end{array}$$

(8) $$\begin{array}{l} \prod_{BC}=\frac{1}{2}\int_{l}\left\{k_{u,0}u_{1}^{2}+k_{v,0}v_{1}^{2}+k_{w,0}w_{1}^{2}+K_{x,0}\psi_{x_{1}}^{2}+K_{\theta ,0}\psi_{\theta_{1}}^{2}\right\}dl\\ +\frac{1}{2}\int_{l}\left\{k_{u,1}u_{N_{L}}^{2}+k_{v,1}v_{N_{L}}^{2}+k_{w,1}w_{N_{L}}^{2}+K_{x,1}\psi_{x_{N_{L}}}^{2}+K_{\theta ,1}\psi_{\theta_{N_{L}}}^{2}\right\}dl\\ +\frac{1}{2}\int_{\theta }\left\{k_{u,2}u_{1}^{2}+k_{v,2}v_{1}^{2}+k_{w,2}w_{1}^{2}+K_{x,2}\psi_{x_{1}}^{2}+K_{\theta ,2}\psi_{\theta_{1}}^{2}\right\}d\theta \\ +\frac{1}{2}\int_{\theta }\left\{k_{u,3}u_{N_{\theta }}^{2}+k_{v,3}v_{N_{\theta }}^{2}+k_{w,3}w_{N_{\theta }}^{2}+K_{x,3}\psi_{x_{N_{\theta }}}^{2}+K_{\theta ,3}\psi_{\theta_{N_{\theta }}}^{2}\right\}d\theta \end{array}$$

On the basis of FSDT, the maximum kinematic energy *T_i,j_*, strain energy *U_i,j_*, and force potential *W_i,j_* are expressed as follows:

(9) $$T_{i,j}=\frac{1}{2}\int_{0}^{L_{i}}\int_{0}^{\theta_{j}}\left\{\begin{array}{l} I_{0}\left[{\left({\dot{u}}_{0}^{i,j}\right)}^{2}+{\left({\dot{v}}_{0}^{i,j}\right)}^{2}+{\left({\dot{w}}_{0}^{i,j}\right)}^{2}\right]+\\ 2I_{1}\left({\dot{u}}_{0}^{i,j}{\dot{\psi }}_{x}^{i,j}+{\dot{v}}_{0}^{i,j}{\dot{\psi }}_{\theta }^{i,j}\right)+I_{2}\left[{\left({\dot{\psi }}_{x}^{i,j}\right)}^{2}+{\left({\dot{\psi }}_{\theta }^{i,j}\right)}^{2}\right] \end{array}\right\}R_{\theta }dxd\theta$$

(10) $$U_{i,j}=\frac{1}{2}\int_{0}^{L_{i}}\int_{0}^{\theta_{j}}\left(N^{T}\cdot Ε+M^{T}\cdot \gamma +Q^{T}Χ\right)R_{\theta }dxd\theta$$

(11) $$W_{i,j}=\frac{1}{2}\int_{0}^{L_{i}}\int_{0}^{\theta_{j}}\left(f_{u_{i,j}}u_{0}^{\left(i.j\right)}+f_{v_{i,j}}v_{0}^{\left(i.j\right)}+f_{w_{i,j}}w_{0}^{\left(i.j\right)}+m_{x_{i,j}}\psi_{x}^{\left(i.j\right)}+m_{\theta_{i,j}}\psi_{\theta }^{\left(i.j\right)}\right)R_{\theta }dxd\theta$$

in which

(12) $$\begin{array}{l} N={\left[\begin{array}{ccc} N_{x} & N_{\theta } & N_{x\theta } \end{array}\right]}^{T},M={\left[\begin{array}{ccc} M_{x} & M_{\theta } & M_{x\theta } \end{array}\right]}^{T},Q={\left[\begin{array}{cc} Q_{x} & Q_{\theta } \end{array}\right]}^{T}\\ Ε={\left[\begin{array}{ccc} \frac{\partial u_{0}}{\partial x} & \frac{1}{R_{\theta }}\left(\frac{\partial v_{0}}{\partial \theta }+w_{0}\right) & \frac{\partial v_{0}}{\partial x}+\frac{1}{R_{\theta }}\frac{\partial u_{0}}{\partial \theta } \end{array}\right]}^{T},\gamma ={\left[\begin{array}{ccc} \frac{\partial \psi_{x}}{\partial x} & \frac{1}{R_{\theta }}\frac{\partial \psi_{\theta }}{\partial \theta } & \frac{\partial \psi_{\theta }}{\partial x}+\frac{1}{R_{\theta }}\frac{\partial \psi_{x}}{\partial \theta } \end{array}\right]}^{T}\\ Χ={\left[\begin{array}{cc} \frac{\partial w_{0}}{\partial x}+\psi_{x} & \frac{1}{R_{\theta }}\left(\frac{\partial w_{0}}{\partial \theta }-v_{0}\right)+\psi_{\theta } \end{array}\right]}^{T},\left[\begin{array}{c} N\\ M \end{array}\right]=\left[\begin{array}{cc} A & B\\ B & D \end{array}\right]\left[\begin{array}{c} Ε\\ \gamma \end{array}\right],Q=CΧ\\ A=\left[\begin{array}{ccc} A_{11} & A_{12} & 0\\ A_{12} & A_{11} & 0\\ 0 & 0 & A_{66} \end{array}\right],B=\left[\begin{array}{ccc} B_{11} & B_{12} & 0\\ B_{12} & B_{11} & 0\\ 0 & 0 & B_{66} \end{array}\right],D=\left[\begin{array}{ccc} D_{11} & D_{12} & 0\\ D_{12} & D_{11} & 0\\ 0 & 0 & D_{66} \end{array}\right],C=\left[\begin{array}{cc} \kappa A_{66} & 0\\ 0 & \kappa A_{66} \end{array}\right], \end{array}$$

Finally, to ensure the accuracy of the numerical calculation, the LSWRM is employed. By introducing weighting parameters Г*_t_* (*t* = *u*, *v*, *w*, *x*, *θ*), Equations (4)–(6) can be rewritten as follows [27]:

(13) $$\bar{\prod }=\int_{t_{0}}^{t_{1}}\sum_{i=1}^{N_{L}}\sum_{j=1}^{N_{\theta }}\left(T_{i,j}-U_{i,j}+W_{i,j}\right)dt+\int_{t_{0}}^{t_{1}}\left(\sum_{i,i+1}\left({\tilde{\prod }}_{\lambda x}-{\tilde{\prod }}_{\kappa x}\right)+\sum_{j,j+1}\left({\tilde{\prod }}_{\lambda \theta }-{\tilde{\prod }}_{\kappa \theta }\right)\right)dt+\int_{t_{0}}^{t_{1}}\prod_{BC}dt$$

(14) $${\tilde{\prod }}_{\lambda x}=\frac{1}{2}\int_{l}\left(\Gamma_{u}\gamma_{u}^{2}+\Gamma_{v}\gamma_{v}^{2}+\Gamma_{w}\gamma_{w}^{2}+\Gamma_{x}\gamma_{x}^{2}+\Gamma_{\theta }\gamma_{\theta }^{2}\right)dl$$

(15) $${\tilde{\prod }}_{\lambda \theta }=\frac{1}{2}\int_{\theta }\left(\Gamma_{u}\zeta_{u}^{2}+\Gamma_{v}\zeta_{v}^{2}+\Gamma_{w}\zeta_{w}^{2}+\Gamma_{x}\zeta_{x}^{2}+\Gamma_{\theta }\zeta_{\theta }^{2}\right)d\theta$$

### 2.3. Allowable Displacement Functions and Corresponding Motion Equations

By applying the MVP to enforce continuity at the interfaces and adhere to boundary conditions, the displacement functions no longer require explicit compliance with the constraints imposed by the internal boundaries and geometric interfaces. As a result, the updated variational functional in Equation (13) permits the use of any set of linearly independent and complete basis functions to represent the displacements. In this work, the displacement functions for the FGP elliptic shells and panels are expressed in terms of Jacobi polynomials, as described in the following form [2]:

(16a) $$u_{0}^{i,j}\left(x,\theta ,t\right)=\sum_{m=0}^{M}\sum_{n=0}^{N}U_{mn}P_{m}^{\left(\alpha ,\beta \right)}(x)P_{n}^{\left(\alpha ,\beta \right)}(\theta )u_{mn}\left(t\right)={\tilde{U}}_{mn}U\left(x,\theta \right)u\left(t\right)$$

(16b) $$v_{0}^{i,j}\left(x,\theta ,t\right)=\sum_{m=0}^{M}\sum_{n=0}^{N}V_{mn}P_{m}^{\left(\alpha ,\beta \right)}(x)P_{n}^{\left(\alpha ,\beta \right)}(\theta )v_{mn}\left(t\right)={\tilde{V}}_{mn}V\left(x,\theta \right)v\left(t\right)$$

(16c) $$w_{0}^{i,j}\left(x,\theta ,t\right)=\sum_{m=0}^{M}\sum_{n=0}^{N}W_{mn}P_{m}^{\left(\alpha ,\beta \right)}(x)P_{n}^{\left(\alpha ,\beta \right)}(\theta )w_{mn}\left(t\right)={\tilde{W}}_{mn}W\left(x,\theta \right)w\left(t\right)$$

(16d) $$\psi_{x}^{i,j}\left(x,\theta ,t\right)=\sum_{m=0}^{M}\sum_{n=0}^{N}\Psi_{mn}P_{m}^{\left(\alpha ,\beta \right)}(x)P_{n}^{\left(\alpha ,\beta \right)}(\theta )\psi_{mn}\left(t\right)={\tilde{\Psi }}_{mn}\Psi \left(x,\theta \right)\psi \left(t\right)$$

(16e) $$\psi_{\theta }^{i,j}\left(x,\theta ,t\right)=\sum_{m=0}^{M}\sum_{n=0}^{N}\Phi_{mn}P_{m}^{\left(\alpha ,\beta \right)}(x)P_{n}^{\left(\alpha ,\beta \right)}(\theta )\varphi_{mn}\left(t\right)={\tilde{\Phi }}_{mn}\Phi \left(x,\theta \right)\varphi \left(t\right)$$

where ${\bar{U}}_{m}$, ${\bar{V}}_{m}$, ${\bar{W}}_{m}$, ${\bar{\Psi }}_{x,m}$ and ${\bar{\Psi }}_{\theta ,m}$ are the generalized coordinate vector. $U\left(x,\theta \right)$, $V\left(x,\theta \right)$, $W\left(x,\theta \right)$, $\Psi_{x}$, and $\Psi_{\theta }\left(x,\theta \right)$ represent the corresponding coefficients for the Jacobi expansion.

$U_{m}$, $V_{m}$, $W_{m}$, $\Psi_{x,m}$, and $\Psi_{\theta ,m}$ are the corresponding Jacobi expanded coefficients. $P_{m}^{\left(\alpha ,\beta \right)}(x)$ denotes the *m*-th order Jacobi polynomial along the length direction. In fact, Jacobi polynomials can be considered an extension of various orthogonal polynomials. By choosing different values for *α* and *β*, they can evolve into well-known polynomials like Legendre and Chebyshev.

By substituting Equations (4)–(6) and (14)–(16) (where Equation (16) is composed of Equation (16a–e)) into Equation (13) and performing variational calculation, the governing equations of the structure can be obtained:

(17) $$M\ddot{q}+\left[K-{\bar{K}}_{\lambda x}-{\bar{K}}_{\lambda \theta }+{\bar{K}}_{\kappa x}+{\bar{K}}_{\kappa \theta }-{\hat{K}}_{B}\right]q=F$$

where **M** and **K** are the mass and stiffness matrices of the structure, respectively, **q** is the displacement coefficient matrix, and **F** is the external force matrix. However, since this paper focuses on the study of free vibration characteristics, **F** is neglected. Assuming harmonic motion, $q=\tilde{q}e^{i\omega t}$, $F=\tilde{F}e^{i\omega t}$, Equation (17) is further transformed into a free vibration characteristic solution equation, which is expressed as follows:

(18) $$\left[\left(K-{\bar{K}}_{\lambda x}-{\bar{K}}_{\lambda \theta }+{\bar{K}}_{\kappa x}+{\bar{K}}_{\kappa \theta }-{\hat{K}}_{B}\right)-\omega^{2}M\right]\tilde{q}=0$$

## 3. Computational Results and Analysis

Based on the MVP, LSWRM, and segmentation technologies, a theoretical model has been established in the previous section to study the dynamic characteristics of the structure. This model involves numerous modeling parameters and Jacobian parameters, which require an initial study of its convergence characteristics to ensure the stability and reliability of the model. Following this, the prediction accuracy of the theoretical model needs to be verified using the correct model and Jacobian parameters. As mentioned in the introduction, research results on these topics have not yet been published. To provide a more comprehensive study, this paper investigates the most common boundary conditions in engineering, including clamped boundaries (C): $k_{u}=k_{v}=k_{w}=K_{x}=K_{\theta }=10^{15}$, elastic boundaries (E): $k_{v}=k_{w}=k_{u}=K_{x}=K_{\theta }\neq 0$, free boundaries (F): $k_{u}=k_{v}=k_{w}=K_{x}=K_{\theta }=0$, shear diaphragm supports (SD): $k_{v}=k_{w}=10^{15},\;k_{u}=K_{x}=K_{\theta }=0$, and simply supported boundaries (SS): $k_{u}=k_{v}=k_{w}=K_{\theta }=10^{15},\;K_{x}=0$. In the following numerical examples, unless otherwise specified, the following geometric parameters are used: shells: *L* = 3 m, *h* = 0.1 m, *a* = 2 m, *b* = 1 m, *θ*_0_ = 360°; panels: *L* = 3 m, *h* = 0.1 m, *a* = 2 m, *b* = 1 m, *θ*_0_ = 120°. The non-dimensional frequency parameter is defined as follows: $\Omega =\omega L^{2}/h\sqrt{\rho_{1}/E_{1}}$.

### 3.1. Convergence and Validation

Table 1 presents the relationship between the Ω and the weighted parameter for FGP elliptic shells and panels under the C-C boundary condition, illustrating the convergence trend. The weight parameter coefficients are uniformly represented by compound *κ*, whose variation ranges from 10^4^ to 10^24^. From Table 1, clearly, when the weight parameter is extremely small, the calculation results deviate significantly from the actual values, indicating that the modal information in this case represents a pseudo-mode. When the stiffness coefficient is between 10^12^ and 10^18^, the calculation results are stable, which is in good agreement with the actual calculation frequency. Then, we can think that the weighted parameter interval can stabilize the numerical results. However, when the weight parameter exceeds 10^22^, we can find that the calculation results have diverged. Therefore, the higher the weight coefficient, the better. For all the following examples, a uniform weight parameter value of 10^16^ is selected.

Table 2 shows the convergence of the Ω versus the boundary parameter in the FGP elliptic shells and panels. By keeping the stiffness of the four springs constant and only varying the stiffness value of one degree of freedom, the boundary convergence is tested. It can be observed that when the spring stiffness values are below 10^8^ or above 10^12^, the results remain almost unchanged. Moreover, the spring stiffness does not necessarily improve with increasing values. For example, when *k_w_*, *K_x_*, *K_θ_* exceeds 10^20^, the frequency results even show a decreasing trend. Therefore, in subsequent calculations, a stiffness value of 10^14^ is chosen for the clamped boundary. In the intermediate region, the general elastic boundary conditions can be simulated equivalently.

**Figure 2:**
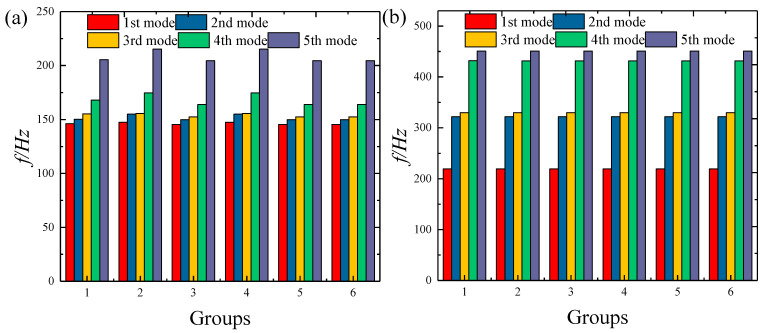
Variation in FGP structure frequencies with different segment numbers: (**a**) elliptic shells; (**b**) elliptic panels.

Figure 2 illustrates how the natural frequencies change as the number of segments varies for the FGP structures with clamped–clamped boundary. Six groups of subsection values are considered in this example, which are as follows: *N_L_* × *N_θ_* = 2 × 2, 2 × 3, 2 × 4, 3 × 3, 3 × 4, 4 × 4. It is clear that the natural frequencies converge rapidly as the number of segments increases, with the frequency results stabilizing once the *N_L_* × *N_θ_* = 3 × 3. However, using an excessively high truncation number substantially increases the computational burden. Therefore, *N_L_* × *N_θ_* = 4 × 4 is used in subsequent calculations.

**Figure 3:**
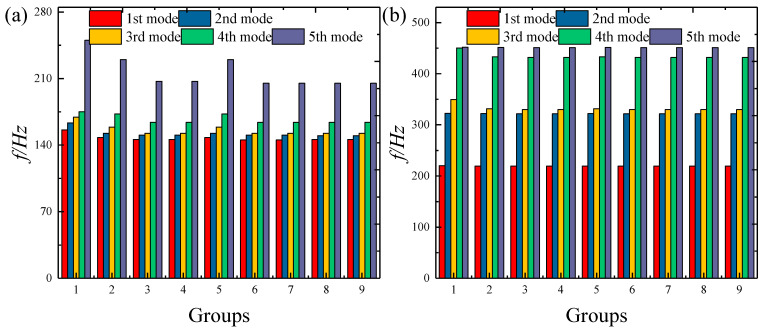
Variation in FGP structure frequencies with different truncation terms: (**a**) elliptic shells; (**b**) elliptic panels.

As mentioned above, the displacement admissible function in this paper is Jacobi polynomials, so it is necessary to study the influence of Jacobian parameters on numerical stability. Figure 3 illustrates the convergence pattern of structural frequencies as influenced by the truncation numbers *M* and *N* of the Jacobi polynomials. Nine groups of truncated value parameters are adopted in this example. Their truncated values are *M* × *N* = 8 × 8, 8 × 10, 8 × 12, 8 × 14, 10 × 10, 10 × 12, 10 × 14, 12 × 12, 14 × 14. This picture clearly shows that when the truncation value exceeds 10 × 12, the numerical results tend to be stable. Therefore, in all subsequent numerical examples, the unified definition is *M* × *N* = 12 × 12. 

Next, the accuracy of the dynamic model is demonstrated. Table 3 provides a comparison of the Ω with those reported in relevant literature, using simply supported boundary conditions. The geometric parameters for the FGP elliptic shell are as follows: *L*/*a* = 0.2, *h*/*a* = 0.01, *a* = *b* = 1 m. The results show an excellent agreement, verifying the reliability of the method presented in this paper.

### 3.2. Numerical Analysis and Parameter Investigations

The convergence and accuracy of the proposed computational method have been validated in the previous section. Next, new numerical results and parametric studies will be carried out in this sub-section. Table 4 and Table 5 show the fundamental frequencies for Type 1 and Type 2 FGP elliptic shells under classical and elastic boundaries. The selected boundary conditions are as follows: C-C, SD-SD, C-F, C-S E1-E1, E2-E2, E3-E3, and E4-E4. The elastic boundary parameters used are set as follows: E1: $k_{v}=k_{u}{=10}^{8},\;k_{w}=K_{x}=K_{\theta }=10^{15}$; E2: $k_{v}=k_{u}=K_{x}=K_{\theta }=10^{15},\;k_{w}{=10}^{8}$; E3: $k_{v}=k_{u}=k_{w}{=10}^{15},\;$$K_{x}=K_{\theta }=10^{8}$; E4: $k_{v}=k_{u}=k_{w}=K_{x}=K_{\theta }=10^{8}$. In addition, three types of elliptic radius ratio, *a*/*b* = 1, 2, 3, are adopted in this example.

**Figure 4:**
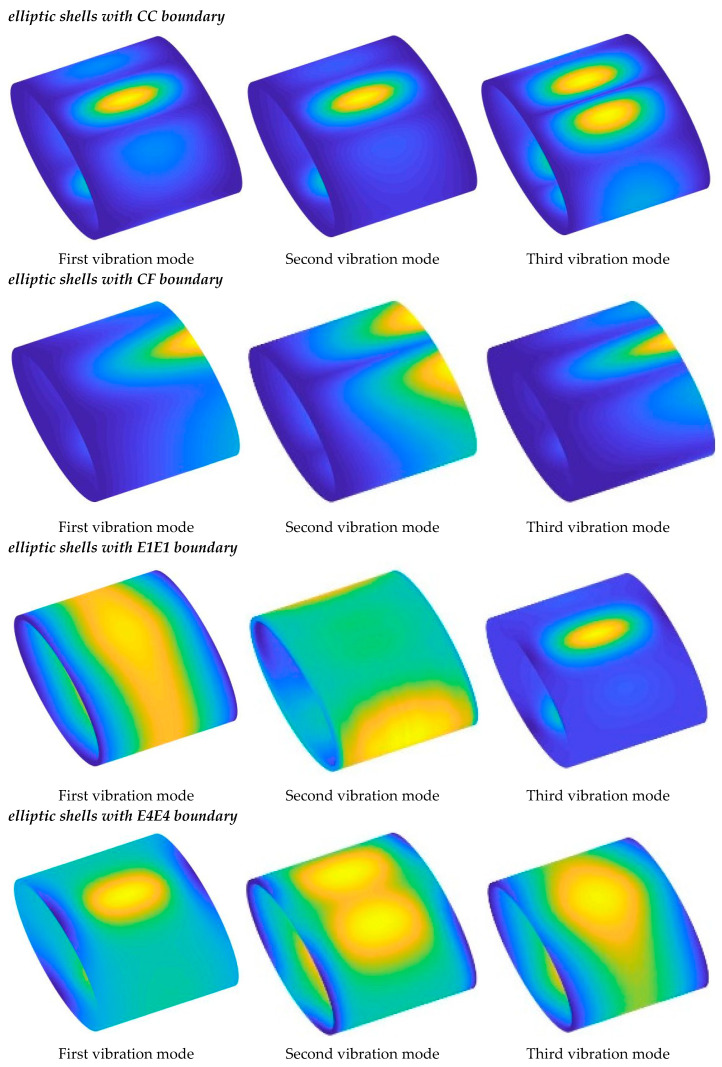
Vibration modes of FGP elliptic shells under various boundary types.

**Figure 5:**
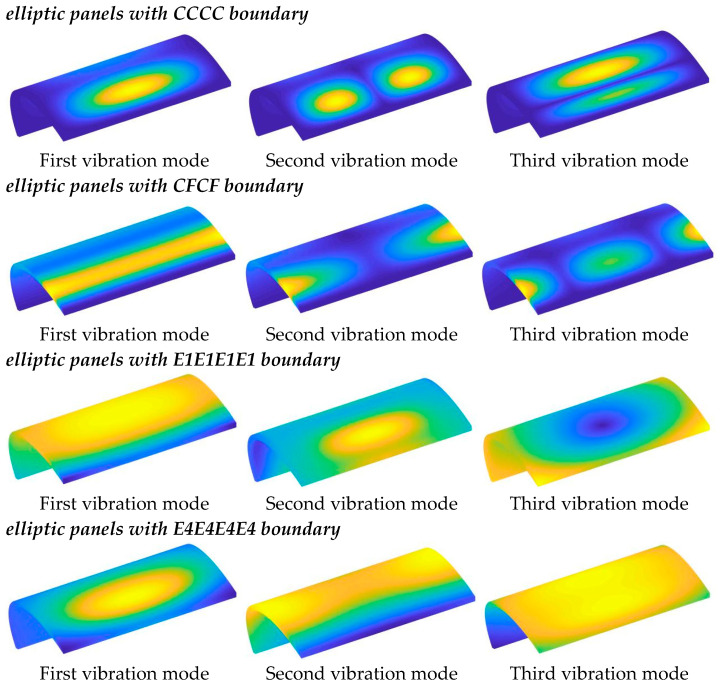
Vibration modes of FGP elliptic panels under various boundary types.

From the results in the two tables, the following conclusions can be drawn: (1) Sensitivity to Boundary Stiffness: The frequency parameters of the structure are highly sensitive to boundary stiffness. Greater boundary stiffness directly correlates with higher frequency results, reflecting the combined stiffness effect of the boundary and the structural stiffness matrix. (2) Effect of Radius Ratio: As the radius ratio increases, the natural frequency of the structure decreases, suggesting that a flatter elliptical shell tends to have lower stability. (3) Influence of Porosity: Generally, an increase in porosity leads to higher natural frequencies, as the increase in porosity parameter raises the structure’s Young’s modulus, enhancing the overall stiffness. Notably, the frequency results for Type 1 are consistently higher than those for Type 2, suggesting that the material parameters used in Type 1 yield a stiffer structure for the selected geometric parameters. Interestingly, different results appear under the E1 and E4 boundary conditions. Observing the parameters, it can be seen that the *k_u_* and *k_v_* values for these boundaries fall within the elastic range, suggesting that these specific spring stiffnesses significantly impact the out-of-plane vibrations of the structure. This leads to complex variations in natural frequencies influenced by both geometric and material parameters. Table 6 and Table 7 present the frequencies of FGP elliptical shells under several classic elastic boundaries. Similarly, Table 8, Table 9, Table 10 and Table 11 display the frequencies of FGP circular plates, where the previously discussed trends are also confirmed. To visually demonstrate the vibration modes of cylindrical shells and panels, the first three modal shapes under various classic boundary conditions are shown in Figure 4 and Figure 5.

**Figure 6:**
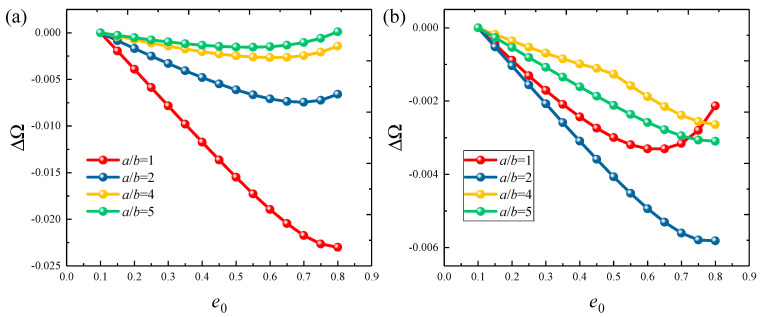

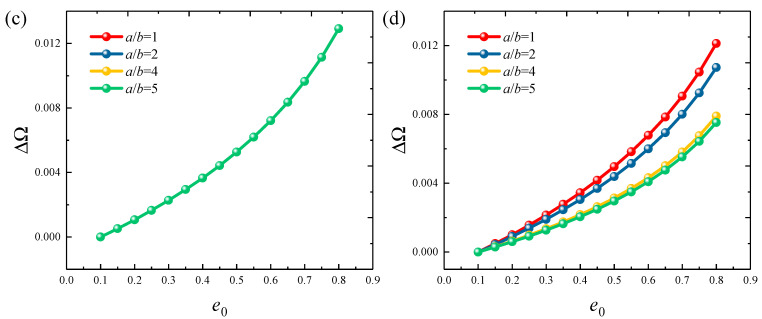
Variation in ΔΩ versus the porosity coefficients *e*_0_ for functionally graded porous elliptic shells: (**a**) C-C; (**b**) C-F; (**c**) E1-E1; (**d**) E4-E4.

**Figure 7:**
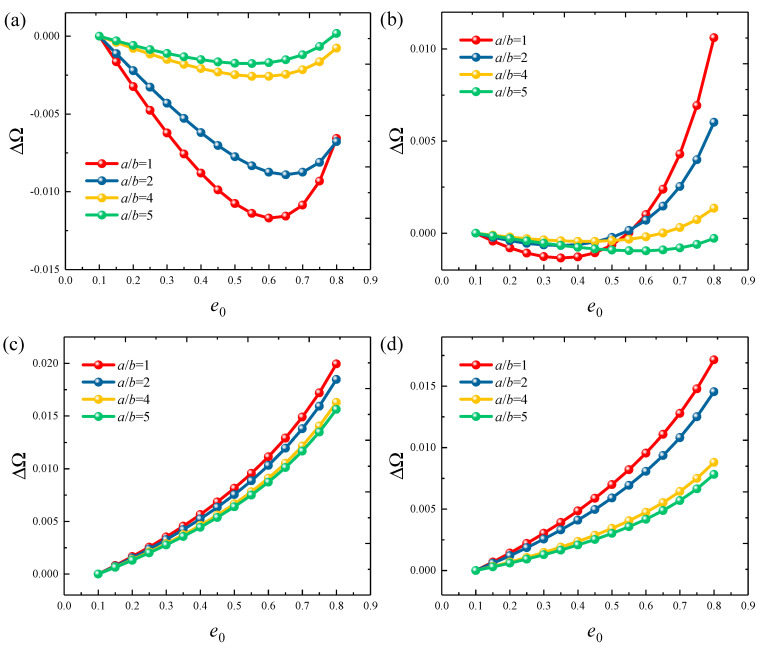
Variation in ΔΩ versus the porosity coefficients *e*_0_ for functionally graded porous elliptic panels: (**a**) CCCC; (**b**) CFCF; (**c**) E1E1E1E1; (**d**) E4E4E4E4.

**Figure 8:**
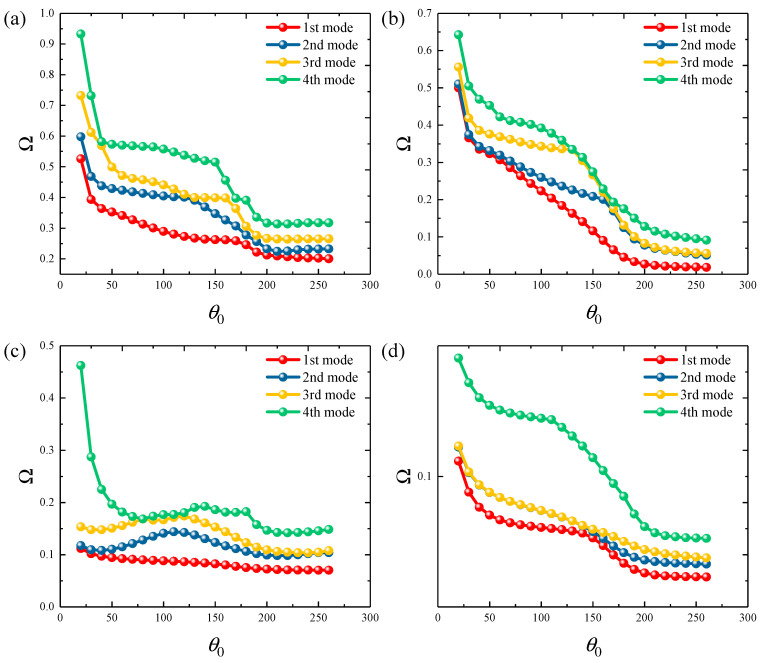
Variation in the frequency parameters ΔΩ versus the circumferential angle *θ*_0_ for functionally graded porous elliptic panels: (**a**) CCCC boundary; (**b**) CFCF boundary; (**c**) E1E1E1E1 boundary; (**d**) E4E4E4E4 boundary.

Figure 6 and Figure 7 show the variation of frequency parameters $\Delta \Omega =\Omega_{\left(e_{0}\right)}-\Omega_{\left(0\right)}$ with porosity parameters *e*_0_ for FGP cylindrical shells and panels under various boundary types, including clamped, free, and several elastic boundaries. The results are quite interesting. Under fixed boundaries, the ΔΩ first decreases and then increases. The porosity parameter at which the frequency of the cylindrical shell changes is higher. However, under elastic boundary conditions, the *e*_0_ and ΔΩ change in sync. This suggests that the *e*_0_ of the structure should be adjusted carefully in different working conditions to achieve optimal dynamic characteristics. Finally, the influence of the circumferential angle *θ*_0_ on the ΔΩ is analyzed, with results presented in Figure 8. The study range is from 20° to 260°. It can be observed that when the *θ*_0_ is below 50°, the ΔΩ decreases significantly with the increasing angle and then slowly increases. For elastic boundary conditions, the trend is more complex.

## 4. Conclusions

This research introduces a comprehensive dynamic model to investigate the free vibration properties of FGP elliptical shells and panels. The motion energy equation of the structure is established based on FSDT and LSWRM. A virtual spring technique is introduced to simulate arbitrary boundary types. The segmentation method and modified variational principle are employed to ensure continuity in the integrals. The admissible displacement functions are constructed using Jacobi polynomials with variable coefficients, and the vibration equations are derived through the Ritz method. The proposed model has been validated, showing high computational accuracy and precision, offering valuable reference data for future researchers. Moreover, parametric studies reveal that the natural frequencies of the structure are highly sensitive to boundary conditions, with larger boundary stiffness contributing to greater dynamic stability. In most cases, the natural frequency negatively correlates with material porosity, although the behavior under elastic boundary conditions is more complex, warranting further investigation into its mechanisms.

## Figures and Tables

**Table 1:** Convergence analysis of the weighted parameter (C-C boundary condition).

*κ*	Elliptic Shells				Elliptic Panels			
	1	2	3	4	1	2	3	4
10^4^	0.0886	0.0887	0.0887	0.0887	0.1146	0.1146	0.2130	0.2131
10^6^	0.0899	0.0920	0.0923	0.0943	0.1167	0.1188	0.2145	0.2170
10^8^	0.1114	0.1612	0.1729	0.1839	0.1689	0.2271	0.2566	0.3300
10^10^	0.1680	0.1805	0.1853	0.2023	0.2615	0.3850	0.3880	0.5019
10^12^	0.1817	0.1868	0.1932	0.2089	0.2727	0.4003	0.4102	0.5370
10^14^	0.1819	0.1869	0.1933	0.2090	0.2729	0.4005	0.4105	0.5375
10^16^	0.1819	0.1870	0.1933	0.2090	0.2729	0.4005	0.4105	0.5375
10^18^	0.1819	0.1870	0.1933	0.2090	0.2729	0.4006	0.4105	0.5375
10^20^	0.1821	0.1871	0.1933	0.2094	0.2704	0.4011	0.4106	0.5347
10^22^	0.1525	0.1779	0.2646	0.2646	0.3255	0.4131	0.5307	0.6446
10^24^	0.4118	0.4118	0.4793	0.5368	0.9096	0.9096	1.0145	1.3892

**Table 2:** Convergence analysis of boundary conditions.

Value	Elliptic Shells					Elliptic Panels				
	*k_u_*	*k_v_*	*k_w_*	*K_x_*	*K_θ_*	*k_u_*	*k_v_*	*k_w_*	*K_x_*	*K_θ_*
10^4^	0.1759	0.1712	0.1759	0.1728	0.1818	0.2700	0.2633	0.2689	0.2668	0.2729
10^6^	0.1759	0.1712	0.1759	0.1729	0.1818	0.2700	0.2633	0.2689	0.2668	0.2729
10^8^	0.1759	0.1714	0.1766	0.1782	0.1819	0.2700	0.2634	0.2692	0.2702	0.2729
10^10^	0.1780	0.1781	0.1813	0.1818	0.1819	0.2708	0.2682	0.2724	0.2728	0.2729
10^12^	0.1818	0.1818	0.1819	0.1819	0.1819	0.2728	0.2728	0.2729	0.2729	0.2729
10^14^	0.1819	0.1819	0.1819	0.1819	0.1819	0.2729	0.2729	0.2729	0.2729	0.2729
10^16^	0.1819	0.1819	0.1819	0.1819	0.1819	0.2729	0.2729	0.2729	0.2729	0.2729
10^18^	0.1819	0.1819	0.1819	0.1818	0.1819	0.2729	0.2729	0.2729	0.2729	0.2729
10^20^	0.1819	0.1819	0.1819	0.1502	0.1819	0.2729	0.2729	0.2729	0.2669	0.2729
10^22^	0.1819	0.1819	0.1819	0.1997	0.1764	0.2729	0.2729	0.2730	0.3605	0.2772
10^24^	0.1819	0.1821	0.1752	0.2641	0.1903	0.2729	0.2730	0.2727	0.4777	0.0883

**Table 3:** Comparison of the first five natural frequencies with the literature results (S-S boundary).

Mode No.	Type 1						Type 2					
	*e*_0_ = 0.2			*e*_0_ = 0.4			*e*_0_ = 0.2			*e*_0_ = 0.4		
	Ref. [20]	Ref. [17]	Present	Ref. [20]	Ref. [17]	Present	Ref. [20]	Ref. [17]	Present	Ref. [20]	Ref. [17]	Present
1	1.2155	1.2161	1.2161	1.1893	1.1900	1.1900	1.2037	1.2043	1.2043	1.1598	1.1606	1.1605
2	1.2118	1.2121	1.2121	1.1862	1.1866	1.1866	1.1997	1.1996	1.1995	1.1559	1.1565	1.1564
3	1.2064	1.2064	1.2063	1.1818	1.1818	1.1817	1.1938	1.1940	1.1939	1.1501	1.1507	1.1505
4	1.2006	1.2002	1.2000	1.1772	1.1768	1.1766	1.1872	1.1871	1.1869	1.1438	1.1441	1.1438
5	1.1958	1.1952	1.1956	1.1740	1.1733	1.1726	1.1815	1.1813	1.1806	1.1382	1.1384	1.1378

**Table 4:** Variation in the first natural frequency of Type 1 FGP elliptic shells under various boundary conditions.

*a*/*b*	*e*_0_	Boundary Types							
		C-C	SD-SD	C-F	C-S	E1-E1	E2-E2	E3-E3	E4-E4
*a*/*b* = 1	0	0.2926	0.2187	0.1158	0.2855	0.0577	0.2834	0.2848	0.0570
	0.2	0.2848	0.2131	0.1140	0.2777	0.0597	0.2756	0.2773	0.0589
	0.4	0.2770	0.2075	0.1125	0.2699	0.0623	0.2677	0.2698	0.0613
	0.8	0.2657	0.2000	0.1128	0.2581	0.0715	0.2559	0.2590	0.0700
*a*/*b* = 2	0	0.1853	0.1525	0.0824	0.1762	0.0577	0.1755	0.1777	0.0553
	0.2	0.1819	0.1500	0.0803	0.1728	0.0597	0.1720	0.1746	0.0570
	0.4	0.1787	0.1477	0.0782	0.1695	0.0623	0.1687	0.1719	0.0592
	0.8	0.1770	0.1473	0.0755	0.1671	0.0715	0.1662	0.1706	0.0669
*a*/*b* = 3	0	0.1279	0.1028	0.0612	0.1158	0.0577	0.1157	0.1199	0.0525
	0.2	0.1256	0.1006	0.0596	0.1134	0.0597	0.1134	0.1180	0.0539
	0.4	0.1236	0.0984	0.0579	0.1111	0.0623	0.1112	0.1162	0.0556
	0.8	0.1227	0.0963	0.0554	0.1090	0.0715	0.1096	0.1158	0.0620

**Table 5:** Variation of the first natural frequency of Type 2 FGP elliptic shells under various boundary conditions.

*a*/*b*	*e*_0_	Boundary Types							
		C-C	SD-SD	C-F	C-S	E1-E1	E2-E2	E3-E3	E4-E4
*a*/*b* = 1	0	0.2926	0.2187	0.1158	0.2855	0.0577	0.2834	0.2848	0.0570
	0.2	0.2829	0.2115	0.1121	0.2753	0.0597	0.2740	0.2750	0.0589
	0.4	0.2723	0.2036	0.1077	0.2639	0.0622	0.2637	0.2643	0.0613
	0.8	0.2485	0.1854	0.0951	0.2376	0.0713	0.2414	0.2406	0.0697
*a*/*b* = 2	0	0.1853	0.1525	0.0824	0.1762	0.0577	0.1755	0.1777	0.0553
	0.2	0.1794	0.1478	0.0797	0.1702	0.0597	0.1700	0.1723	0.0570
	0.4	0.1727	0.1423	0.0768	0.1634	0.0622	0.1638	0.1661	0.0591
	0.8	0.1544	0.1267	0.0700	0.1448	0.0714	0.1477	0.1495	0.0661
*a*/*b* = 3	0	0.1279	0.1028	0.0612	0.1158	0.0577	0.1157	0.1199	0.0525
	0.2	0.1239	0.0996	0.0593	0.1121	0.0597	0.1124	0.1167	0.0538
	0.4	0.1193	0.0960	0.0571	0.1079	0.0623	0.1089	0.1131	0.0553
	0.8	0.1068	0.0868	0.0524	0.0972	0.0714	0.1000	0.1032	0.0598

**Table 6:** Fundamental frequencies for Type 1 functionally graded porous elliptic shells under various boundary conditions.

*a*/*b*	*e*_0_	Boundary Types							
		C-E1	C-E2	C-E3	C-E4	SD-E1	SD-E2	SD-E3	SD-E4
*a*/*b* = 1	0	0.2598	0.2879	0.2886	0.1550	0.0407	0.2509	0.2515	0.0407
	0.2	0.2534	0.2801	0.2810	0.1547	0.0422	0.2442	0.2450	0.0421
	0.4	0.2471	0.2723	0.2733	0.1548	0.0440	0.2375	0.2384	0.0440
	0.8	0.2390	0.2607	0.2623	0.1604	0.0505	0.2278	0.2291	0.0505
*a*/*b* = 2	0	0.1716	0.1800	0.1813	0.1120	0.0407	0.1644	0.1653	0.0407
	0.2	0.1686	0.1766	0.1781	0.1115	0.0422	0.1614	0.1625	0.0421
	0.4	0.1659	0.1734	0.1752	0.1112	0.0440	0.1586	0.1599	0.0440
	0.8	0.1646	0.1711	0.1737	0.1142	0.0505	0.1570	0.1588	0.0505
*a*/*b* = 3	0	0.1191	0.1206	0.1237	0.0870	0.0407	0.1094	0.1108	0.0407
	0.2	0.1172	0.1182	0.1216	0.0866	0.0422	0.1071	0.1087	0.0421
	0.4	0.1155	0.1161	0.1197	0.0865	0.0440	0.1050	0.1066	0.0440
	0.8	0.1155	0.1148	0.1191	0.0891	0.0505	0.1030	0.1051	0.0505

**Table 7:** Fundamental frequencies for Type 2 functionally graded porous elliptic shells under various boundary conditions.

*a*/*b*	*e*_0_	Boundary Types							
		C-E1	C-E2	C-E3	C-E4	SD-E1	SD-E2	SD-E3	SD-E4
*a*/*b* = 1	0	0.2598	0.2879	0.2886	0.1550	0.0407	0.2509	0.2515	0.0407
	0.2	0.2513	0.2784	0.2789	0.1530	0.0421	0.2426	0.2431	0.0421
	0.4	0.2418	0.2679	0.2682	0.1507	0.0439	0.2336	0.2338	0.0439
	0.8	0.2195	0.2449	0.2445	0.1456	0.0503	0.2133	0.2129	0.0503
*a*/*b* = 2	0	0.1716	0.1800	0.1813	0.1120	0.0407	0.1644	0.1653	0.0407
	0.2	0.1662	0.1744	0.1757	0.1108	0.0421	0.1593	0.1602	0.0421
	0.4	0.1600	0.1680	0.1693	0.1096	0.0439	0.1534	0.1543	0.0439
	0.8	0.1428	0.1509	0.1519	0.1078	0.0503	0.1375	0.1382	0.0503
*a*/*b* = 3	0	0.1191	0.1206	0.1237	0.0870	0.0407	0.1094	0.1108	0.0407
	0.2	0.1154	0.1171	0.1201	0.0861	0.0421	0.1061	0.1075	0.0421
	0.4	0.1112	0.1131	0.1161	0.0851	0.0439	0.1025	0.1038	0.0439
	0.8	0.0991	0.1030	0.1049	0.0827	0.0504	0.0933	0.0942	0.0504

**Table 8:** Fundamental frequencies for Type 1 functionally graded porous elliptic panels under various boundary conditions.

*a*/*b*	*e*_0_	Boundary Types							
		CCCC	SDSDSDSD	CFCF	CSCS	E1E1E1E1	E2E2E2E2	E3E3E3E3	E4E4E4E4
*a*/*b* = 1	0	0.4641	0.2727	0.1324	0.3887	0.0898	0.3471	0.4195	0.0858
	0.2	0.4574	0.2685	0.1300	0.3807	0.0929	0.3433	0.4139	0.0885
	0.4	0.4519	0.2594	0.1280	0.3732	0.0969	0.3406	0.4094	0.0920
	0.8	0.4541	0.2434	0.1273	0.3671	0.1112	0.3313	0.4126	0.1043
*a*/*b* = 2	0	0.2774	0.1669	0.1056	0.2455	0.0835	0.2155	0.2577	0.0773
	0.2	0.2729	0.1649	0.1044	0.2406	0.0864	0.2125	0.2538	0.0796
	0.4	0.2689	0.1632	0.1035	0.2359	0.0901	0.2098	0.2504	0.0825
	0.8	0.2683	0.1651	0.1051	0.2322	0.1033	0.2094	0.2505	0.0930
*a*/*b* = 3	0	0.1704	0.1093	0.0902	0.1557	0.0781	0.1401	0.1580	0.0674
	0.2	0.1678	0.1078	0.0895	0.1530	0.0808	0.1385	0.1559	0.0691
	0.4	0.1656	0.1065	0.0891	0.1507	0.0843	0.1372	0.1542	0.0713
	0.8	0.1659	0.1072	0.0916	0.1500	0.0966	0.1383	0.1551	0.0793

**Table 9:** Fundamental frequencies for Type 2 functionally graded porous elliptic panels under various boundary conditions.

*a*/*b*	*e*_0_	Boundary Types							
		CCCC	SDSDSDSD	CFCF	CSCS	E1E1E1E1	E2E2E2E2	E3E3E3E3	E4E4E4E4
*a*/*b* = 1	0	0.4641	0.2727	0.1324	0.3887	0.0898	0.3471	0.4195	0.0858
	0.2	0.4490	0.2641	0.1282	0.3777	0.0929	0.3374	0.4088	0.0884
	0.4	0.4313	0.2535	0.1234	0.3653	0.0968	0.3262	0.3964	0.0916
	0.8	0.3787	0.2185	0.1102	0.3347	0.1108	0.2947	0.3597	0.1024
*a*/*b* = 2	0	0.2774	0.1669	0.1056	0.2455	0.0835	0.2155	0.2577	0.0773
	0.2	0.2686	0.1618	0.1024	0.2392	0.0863	0.2110	0.2512	0.0794
	0.4	0.2585	0.1557	0.0986	0.2322	0.0900	0.2063	0.2437	0.0820
	0.8	0.2294	0.1369	0.0865	0.2148	0.1030	0.1969	0.2220	0.0899
*a*/*b* = 3	0	0.1704	0.1093	0.0902	0.1557	0.0781	0.1401	0.1580	0.0674
	0.2	0.1652	0.1060	0.0876	0.1517	0.0808	0.1377	0.1543	0.0688
	0.4	0.1590	0.1021	0.0843	0.1472	0.0842	0.1352	0.1498	0.0704
	0.8	0.1411	0.0905	0.0734	0.1349	0.0964	0.1284	0.1365	0.0744

**Table 10:** Fundamental frequencies for Type 1 functionally graded porous elliptic panels under various boundary conditions.

*a*/*b*	*e*_0_	Boundary Types							
		CE1CE1	CE2CE2	CE3CE3	CE4CE4	SDE1SDE1	SDE2SDE2	SDE3SDE3	SDE4SDE4
*a*/*b* = 1	0	0.2843	0.3619	0.4248	0.1970	0.0689	0.3301	0.3985	0.0689
	0.2	0.2770	0.3580	0.4190	0.1971	0.0714	0.3273	0.3935	0.0714
	0.4	0.2696	0.3551	0.4142	0.1981	0.0744	0.3258	0.3896	0.0744
	0.8	0.2594	0.3443	0.4169	0.2081	0.0854	0.3225	0.3936	0.0854
*a*/*b* = 2	0	0.2279	0.2476	0.2634	0.1569	0.0605	0.2280	0.2447	0.0605
	0.2	0.2246	0.2430	0.2593	0.1567	0.0626	0.2241	0.2409	0.0626
	0.4	0.2216	0.2385	0.2556	0.1571	0.0653	0.2202	0.2375	0.0653
	0.8	0.2205	0.2342	0.2553	0.1634	0.0750	0.2164	0.2372	0.0750
*a*/*b* = 3	0	0.1518	0.1659	0.1649	0.1260	0.0529	0.1478	0.1462	0.0529
	0.2	0.1500	0.1629	0.1625	0.1256	0.0548	0.1448	0.1438	0.0548
	0.4	0.1487	0.1602	0.1604	0.1256	0.0571	0.1419	0.1417	0.0571
	0.8	0.1507	0.1587	0.1610	0.1296	0.0655	0.1391	0.1411	0.0655

**Table 11:** Fundamental frequencies for Type 2 functionally graded porous elliptic panels under various boundary conditions.

*a*/*b*	*e*_0_	Boundary Types							
		CE1CE1	CE2CE2	CE3CE3	CE4CE4	SDE1SDE1	SDE2SDE2	SDE3SDE3	SDE4SDE4
*a*/*b* = 1	0	0.2843	0.3619	0.4248	0.1970	0.0689	0.3301	0.3985	0.0689
	0.2	0.2756	0.3516	0.4141	0.1951	0.0713	0.3211	0.3888	0.0713
	0.4	0.2662	0.3396	0.4015	0.1932	0.0744	0.3105	0.3774	0.0744
	0.8	0.2472	0.3055	0.3645	0.1890	0.0851	0.2793	0.3423	0.0851
*a*/*b* = 2	0	0.2279	0.2476	0.2634	0.1569	0.0605	0.2280	0.2447	0.0605
	0.2	0.2209	0.2408	0.2566	0.1551	0.0626	0.2220	0.2386	0.0626
	0.4	0.2126	0.2332	0.2486	0.1531	0.0653	0.2154	0.2314	0.0653
	0.8	0.1879	0.2146	0.2256	0.1473	0.0747	0.1990	0.2102	0.0747
*a*/*b* = 3	0	0.1518	0.1659	0.1649	0.1260	0.0529	0.1478	0.1462	0.0529
	0.2	0.1472	0.1608	0.1604	0.1239	0.0548	0.1432	0.1424	0.0548
	0.4	0.1417	0.1550	0.1552	0.1213	0.0571	0.1381	0.1380	0.0571
	0.8	0.1247	0.1388	0.1397	0.1124	0.0654	0.1245	0.1252	0.0654

## Data Availability

The original contributions presented in this study are included in the article. Further inquiries can be directed to the corresponding author.
